# Socioeconomic Status and Morbidity Rate Inequality in China: Based on NHSS and CHARLS Data

**DOI:** 10.3390/ijerph16020215

**Published:** 2019-01-14

**Authors:** Yunyun Jiang, Haitao Zheng, Tianhao Zhao

**Affiliations:** 1 School of Economics, Peking University, Beijing 100871, China; jiangyunyun@pku.edu.cn; 2 School of Economics and Management, Beihang University, Beijing 100083, China; zhaotianhaobuaasem@163.com

**Keywords:** socioeconomic Status, two-week incidence rate, number of sick days per thousand people, prevalence of chronic diseases

## Abstract

Previous studies have shown there are no consistent and robust associations between socioeconomic status and morbidity rates. This study focuses on the relationship between the socioeconomic status and the morbidity rates in China, which helps to add new evidence for the fragmentary relationship between socioeconomic status and morbidity rates. The *National Health Services Survey* (NHSS) and *China Health and Retirement Longitudinal Study* (CHARLS) data are used to examine whether the association holds in both all-age cohorts and in older only cohorts. Three morbidity outcomes (two-week incidence rate, the prevalence of chronic diseases, and the number of sick days per thousand people) and two socioeconomic status indicators (income and education) are mainly examined. The results indicate that there are quadratic relationships between income per capita and morbidities. This non-linear correlation is similar to the patterns in European countries. Meanwhile, there is no association between education years and the morbidity in China, i.e., either two-week incidence rate or prevalence rate of chronic diseases has no statistically significant relationship with the education level in China.

## 1. Introduction

So far, an abundance of research has been undertaken on the relationship between socioeconomic status and mortality rates. These studies were carried out in the United States [1,2], Canada [3,4], Europe [5,6], and China [7,8], where they found that the people with socioeconomical disadvantages usually have higher mortality rates than those who have higher education or income level. However, mortality is not the only one which matters. Morbidity, which is another basic element of health, is, at least, equally important as mortality [9]. Morbidity has a vital impact on life expectancy and the length of dependent life [10] when it has a different geographic distribution from the distribution of mortality. Specific in China, morbidity in different regions of China varies widely: 2008 statistics show that the Chengguan District in Tibet has a minimal two-week incidence rate of 5.2%, while the Dongcheng District in Beijing has a maximal two-week incidence rate of 53.2%. As for the prevalence of chronic diseases, the Chengguan District in Tibet has a minimal rate of 5.4%, while the Luwan District in Shanghai, however, has a maximum of 33.6%.

Studies tried to explain how income and education level relate to mortality rates. Those who are wealthy and highly educated are less likely to die younger, as they tend to enjoy advantaged access to health-enhancing resources [11,12,13,14,15,16,17,18], and are more likely to live in well-built houses situated in safe neighborhoods in a non-toxic environment [19,20,21,22]. In addition to affording the cares of better quality [23,24,25], people who have a greater socioeconomic status tend to better understand and follow the instructions given by their health care providers [21,26]. Similarly, people who have a better socioeconomic status may also more easily adopt healthy lifestyles [25,27,28], which decreases their exposures to material deprivation and stressful psychosocial environments [25,29,30,31].

However, while the inverse effects of socioeconomic factors on mortality have been reported by a number of studies, no consistent and robust associations have been found between socioeconomic status and morbidity rates [4,9]: A higher income levels experienced lower levels of morbidity in England [32,33], European countries [9], and Nordic countries [34], while no association between income and morbidity was observed in Canada [4]. Meanwhile, a higher education level experienced lower levels of morbidity in the United States [33] and European countries [9,35], while no association between education and morbidity was observed in Canada [4], England [32], and Nordic countries [34]. Nevertheless, in China, the relationship between socioeconomic status and the overall morbidity rates was less focused on: Though a research has found no wealth and education gradients in the prevalence of hypertension [36], and another found there is a lack of socioeconomic gradients in the overall incidence of non-hospitalized injuries for children in China [37]. Therefore, it becomes important to understand and discuss the morbidity rate itself. It is not only because the lack of literature but also because that simple associations between mortality and morbidity cannot be made, nor can the trend of morbidity be inferred from the trend of mortality because of the development of vaccinations and medical technology [38]. We cannot directly apply the conclusions about mortality in China to morbidity in China, where morbidity also has an important impact on people’s life expectancy in China.

This study aims to focus on the relationship between socioeconomic status and the morbidity rates in China, which helps to add new evidence for the fragmentary relationship between socioeconomic status and morbidity [9]. There are three main contributions of this paper. First, we examine the relationship between socioeconomic status and morbidity rates in China. Previous research mainly focused on other countries except for China. Second, both all-age cohorts and older only aged cohorts’ socioeconomic status and morbidity rate are examined in China. Attention was given to the relationship between socioeconomic status and health at all ages [1,2,4,33,39] and older only ages [3,7,8,9,25]. It is a meaningful observation for China to examine the relationship between socioeconomic status and morbidity still hold only in old aging cohorts after checking all the age cohorts. We use two data sources: NHSS (*National Health Services Survey*) questionnaire collects data from Chinese residents at all ages; CHARLS (*China Health and Retirement Longitudinal Study*) questionnaire collects data from Chinese residents aged 45 and older. Third, we discover the non-linearity in the association between income and the morbidity rate in China by incorporating the quadratic term of income into the regression model. Previous studies [9,32] have found the non-linear relationship between income and morbidity, we further examine the existence of the quadratic relationship between income and morbidity.

In summary, this study provides a detailed analysis of the relationship between socioeconomic status and morbidity in China at all ages and old only ages by using three morbidity indicators and two socioeconomic statuses. The structure of the paper is as follows: in Section 2, we describe the data used in our empirical analysis then outline the model; we present empirical results in Section 3; finally, Section 4 contains our conclusion.

## 2. Data and Methods

### 2.1. Data Sources

Our data come from the *National Health Services Survey* (NHSS) in China, and the *China Health and Retirement Longitudinal Study* (CHARLS) [40]. The NHSS survey began in 1993 and is conducted every five years. In this paper, we use the data in 1998, 2003, and 2008. The data of NHSS in 1993, 2013 and later are not used because some important socioeconomic variables were not collected in 1993, and the detailed data of 2013 and later have not been published by the time we conduct this research. As for the CHARLS survey, we use the Harmonized CHARLS data (Version C) published in April 2018. The harmonized CHARLS data contains the data from wave 1 (2011) to wave 4 (2015). We use the data of 2011, 2013, and 2015 surveys which collected consistent variables with which we are concerned.

The NHSS is a national sample survey, and its respondents are the actual population of selected households. A household is defined as the person or people in the same dwelling, regardless of whether family members and others live together or individuals living alone. A multistage stratified random cluster sampling method was adopted in the NHSS. One hundred and fifty-six counties (cities or districts) from 31 provinces, autonomous regions, and municipalities were randomly selected, from which five sample townships (or neighborhoods) were collected, totaling 780 nationwide. In each township (neighborhood), two administrative villages (or neighborhood committees)—1560 across the whole country—were collected. Furthermore, 60 households from each sample village (or neighborhood committee) were randomly selected for further analysis, coming to a total of 93,600 households (nearly 300,000 people) from all over China.

The questionnaires of the NHSS covered: (1) The health service needs of urban and rural residents, including a survey of population and socioeconomic characteristics and health status; (2) health service demands and the utilization of urban and rural areas residents, including treatment of illness, satisfaction rate and reasons for dissatisfaction, public health, maternal and child care, emergency and inpatient service, the utilization of hospital services, and personal payment of medical expenses; (3) urban and rural health security, including health insurance and the composition of the medical security system; and (4) residents’ satisfaction, including satisfaction with the service systems, service delivery, and coverage and level of health insurance.

CHARLS is a nationally representative, multi-disciplinary and public dataset. It covers many aspects from across the interviewees’ lifetimes, including the household, income, health, finance, social security, and so forth. This study used the harmonized CHARLS (Version C), which contains 25,504 observations or rows. The national baseline survey comprises information on about 17,000 individuals and 10,000 households. Our reasons for choosing the CHARLS data are: First, the CHARLS includes detailed information on individuals’ socioeconomic circumstances and prevalence of chronic diseases; second, it collects data from the persons over 45 years old, which will provide data to check whether the association between socioeconomic status and morbidity still hold in older age cohorts.

### 2.2. Variables

Table 1 displays the definitions of the original variables selected from the NHSS data. In the NHSS data, health indicators of morbidity rates includes: cut down in daily activities due to a physical or mental problem [9,41] and long-term disability [9], incidence of severe illness [42], number of bedridden days [42], multimorbidity [39,43], chronic medical morbidity [44], etc. Considering the availability of data, three health outcomes are taken as our dependent variables: two-week incidence rate (*illnessratio*), number of sick days per thousand people (*illnessday*), and prevalence of chronic diseases (*chronicratio*). The two-week incidence rate measures the respondents’ feelings regarding disease, mainly from the perspective of health services. The rate has three outcomes according to reaction to sickness in two weeks: receiving medical treatment in a health institution, taking medicine or some other adjuvant therapy by themselves, and resting for at least one day without receiving medical treatment or taking medicine. The number of sick days is defined as the average number of sick days in two weeks per 1000 surveyed people, which measures the severity of illness. It is highly correlated with the two-week incidence rate, with a correlation coefficient of 0.946. The prevalence of chronic diseases (*chronicratio*) refers to the prevalence of chronic diseases among the surveyed population. The variable is positively related to the other two, with both correlation coefficients being over 0.7.

The variables on socioeconomic status used frequently includes income [3,41,45], education [3,41], occupational prestige [3], and housing tenure [46]. We focus on income and education in this paper. Real income per capita (*income*) is defined as the average annual income per capita, deflated by the GDP deflators of each city to its 1998 purchasing value. The deflator data are from the *China Statistical Yearbook for Regional Economy* (1998–2008). The weighted education years (*edu*) is calculated by years of completed education composition. In addition, several kinds of controlling variables are usually considered in literature: demographic factors [41], consumption and health expenses level [42,47], accessibility and affordability of health services [48,49], and environment factor [19]. The demographic factors in this paper are measured by four variables: the average age of all age group weighted by group size (*average*), the population over 65 years old (*age65*), male population proportion (*male*), and a dummy variable of urbanization (*urban*). Consumption and health service characteristics are measured by the following indicators: total annual consumptions per capita (*expend*), proportion of family health expenditure to total living expenses (*mediratio*), average annual medical treatment cost (*permedicost*) and average hospitalization expense of each time (*perhospitalcost*). The accessibility of health services has two perspectives: geographical accessibility and economic accessibility. The first one considers the distance from and the time cost to medical institutions which measure the physical convenience of accessing health services. Economic accessibility measures people’s capacity to afford medical bills, i.e., a patient’s income level and whether he/she has medical insurance [48]. The accessibility and affordability of health services are measured by the following indicators: proportion of the population within 1 km of the nearest medical and health unit (*distance*), proportion of the population whose time cost to the nearest hospital is less than 10 min (*time10*), and proportion of the population with medical insurance (*insurance*). The environment factor is shown by the proportion of hygienic toilets (*washroom*).

In this paper, the variables from the Harmonized CHARLS data are selected then aggregated to correspond with the variables selected from NHSS data. Table A1 in Appendix A displays the definitions of aggregated variables. Corresponding to the dependent variables in the NHSS dataset, we use the prevalence of chronic diseases (*CHRONIC_RATIO*) as our dependent variable in the analysis on the CHARLS dataset. Meanwhile, the earned income per capita (*AVGINDIINCOME_EARN*) and average education years (*AVGEDU*) are the two main socioeconomic variables that we study in the CHARLS dataset. All variables in the CHARLS dataset can be roughly classified to eight sub-categories: morbidity, income, education, demographic backgrounds, health expenditure, health insurance, job status, and family relationships. The first six categories separately correspond to the variables selected from NHSS data. However, because the respondents of CHARLS are older people who possibly have been retired or been receiving extra financial support from other family members, we add the variables of the last two categories to control the effects of non-earned incomes, such as the transfer payments from children.

### 2.3. Descriptive Statistics

In this paper, we use two-panel datasets to perform econometric analysis: a 3-year (1998, 2003, 2008) panel dataset of NHSS, and a 3-year (2011, 2013, 2015) panel dataset of CHARLS. The NHSS dataset was aggregated by the National Health Commission of China and published at the county level. It covers around 95 counties every year. Meanwhile, the CHARLS dataset was aggregated from the Harmonized CHARLS dataset to city level, where 126 cities are included.

In addition to simplify aggregating variables, we also adjusted the variables of income: the real income per capita (*income*) in the NHSS dataset and the average earned income per capita (*AVGINDIINCOME_EARN*) in the CHARLS dataset. In the NHSS dataset, because of the collinearity (correlation coefficient is 0.639) between the real income per capita and weighted education years, the original real income per capita was firstly regressed by the weighted education years with OLS (ordinary least squares). Then, it was replaced by the regression residuals. However, considering the lower correlation coefficient (0.532) between the earned income per capita and education years in the CHARLS dataset, we did not adjust the earned income per capita. Then, to discover whether there is a quadratic relationship between income per capita and health outcomes, we separately added the squared real income per capita (*income2*) to our NHSS dataset, and the squared earned income per capita (*AVGINDIINCOME_EARN2*) to the CHARLS dataset. Table 2 displays the general descriptive statistics of health outcome indicators, income indicators, and education indicators of the NHSS and CHARLS datasets. Because we used principle component analysis (PCA) to aggregate and rotate our candidate variables to orthogonal components, their descriptive statistics are not reported. In Section 2.4, we introduce how we performed the PCA.

To discover the existence of the inequality of health outcomes, we compute the Gini coefficients of health outcome indicators at county/city level: two-week incidence rate, number of sick days per thousand people, the prevalence of chronic diseases in the NHSS dataset, and the prevalence of chronic diseases in the CHARLS dataset. Table 3 reports the Gini coefficients in every year. The Gini coefficients of the three indicators in the NHSS dataset reveal a moderate inequality of health outcomes. However, the prevalence of chronic diseases in the CHARLS dataset shows a lack of variance. It is partly because that the mean value of the prevalence (75%) is much higher than its counterpart (16.35%) in the NHSS dataset: If most people have at least one kind of chronic diseases, their differences of whether to have a chronic disease lead to a smaller variance in numbers. In addition to Gini coefficients, we also compute other types of inequality indices as a reference. Note: "prop" indicates proportions, a digit in the range from 0 to 1

Table A2 displays the complete tabular, where we can also find the existence of similar inequality of health outcomes.

**Figure 1:**
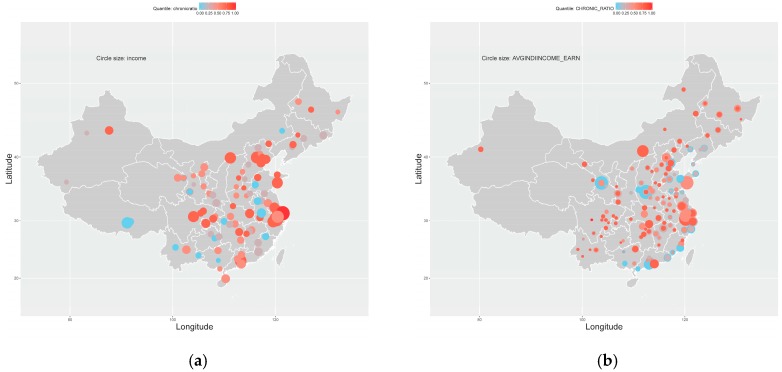
Geographic distributions: income and the prevalence of chronic diseases. (**a**) NHSS; (**b**) CHARLS.

The inequality of health outcomes is related to the difference among counties/cities. For example, Figure 1 displays this relationship using the prevalence of chronic diseases. In Figure 1, every circle marks a county (NHSS) or city (CHARLS) whose X-Y coordinates are defined with real longitudes and latitudes. The circles’ color indicates the relative level of a county’s or a city’s prevalence of chronic diseases when compared with other counties or cities. The red color becomes deeper with the chronic disease prevalence increasing. To make the two sub-figures comparable, we dyed these circles in quantile measure rather than the absolute values of the prevalence of chronic diseases, i.e., a circle’s color indicates the county’s or city’s relative level of chronic disease prevalence in the whole sample. In Figure 1, both sub-figures show a consistent geographic inequality of chronic disease prevalence among counties/cities, e.g., coastal metropolises such as Shanghai (N31.23°, E121.47°) and Guangzhou (N23.13°, E113.27°) have a relatively higher prevalence of chronic diseases; and even counties/cities in the same province may have different levels of chronic disease prevalence. However, this kind of descriptive inequality among counties/cities cannot finally answer whether the inequality of the prevalence of chronic diseases is significantly related to the counties/cities themselves.

**Figure 2:**
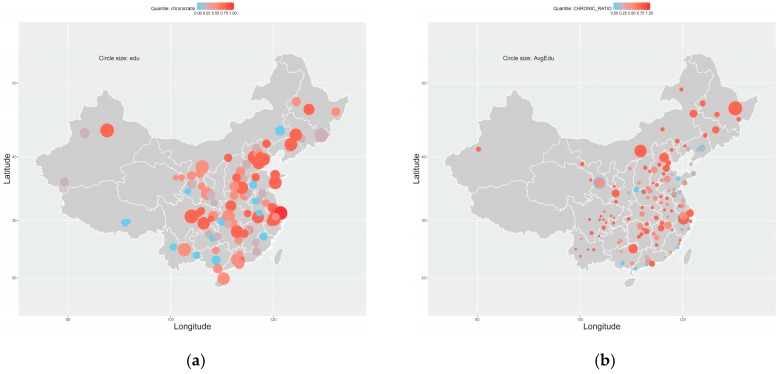
Geographic distributions: average education years and the prevalence of chronic diseases. (**a**) NHSS; (**b**) CHARLS.

In addition to counties/cities themselves, the inequality of chronic disease prevalence is also descriptively related to the income per capita in counties/cities. In Figure 1, circle size denotes the level of income per capita. A larger circle means the county/city has a higher average income level. When combing the circles’ color and size, Figure 1 displays an intuitive pattern that counties/cities with a higher level of income per capita tend to have a relatively higher prevalence of chronic diseases. cities with higher income level, such as Shanghai, Beijing (N39.90°, E116.40°), and Chengdu (N30.67°, E104.07°), usually have higher chronic disease prevalence. However, this pattern is not universal, e.g., Luoyang (N36.03°, E103.73°) and Lanzhou (N36.07°, E103.82°), in the sub-figure of CHARLS dataset have higher incomes per capita but a relatively lower prevalence of chronic diseases. Meanwhile, similar patterns can be observed in Figure 2, where circles’ color and size separately denote chronic disease prevalence and average education years. In the first sub-figure of Figure 2, counties with higher average education years tend to have a higher prevalence of chronic diseases, while this pattern is less intuitive in the second sub-figure. Nevertheless, all these possible correlations are descriptive but without statistical proof: no individual difference considered, no other socioeconomic factors controlled, etc. Therefore, further econometric analysis is required to accurately answer whether there are significant correlations between health outcomes and income per capita or education years.

### 2.4 Model Specification

In this paper, we use panel data models with individual effects as our benchmark models. Because individual difference needs to be controlled to evaluate the real correlations between health outcomes and main socioeconomic status indicator variables such as income per capita and education years. On the one hand, we use panel datasets of both NHSS and CHARLS data. On the other hand, the descriptive statistics, e.g., Figure 1, display possible individual differences at county/city level. Nevertheless, there are 95 counties from 31 provinces in the NHSS dataset and 126 cities from 28 provinces in the CHARLS dataset, while only three years’ data are acquirable. The incidental parameter problem [50] suggested we use province as individual fixed effects and county/city for individual random effects to obtain consistent estimates. In this context, Hausman tests are no longer required if the results of different model specifications are consistent and robust.

Through principle component analysis (PCA), the socioeconomic indicator variables other than income per capita and education years are converted to orthogonal components which are used as control variables in our regression analysis. There are two reasons not to use original socioeconomic indicator variables: One is that many similar variables can together describe a specific aspect of socioeconomic status, e.g., consumptions can be described by both the average amount of annual food consumptions and Engel’s coefficient; the other one is that similar or related socioeconomic indicator variables usually have severe collinearity. Selecting original socioeconomic indicator variables are arbitrary and may result in the failure of estimation. Meanwhile, the trading-off among similar but different variables may also result in omitted variable bias. Therefore, we use dimensionality reduction techniques to solve the problems.

**Figure A1:**
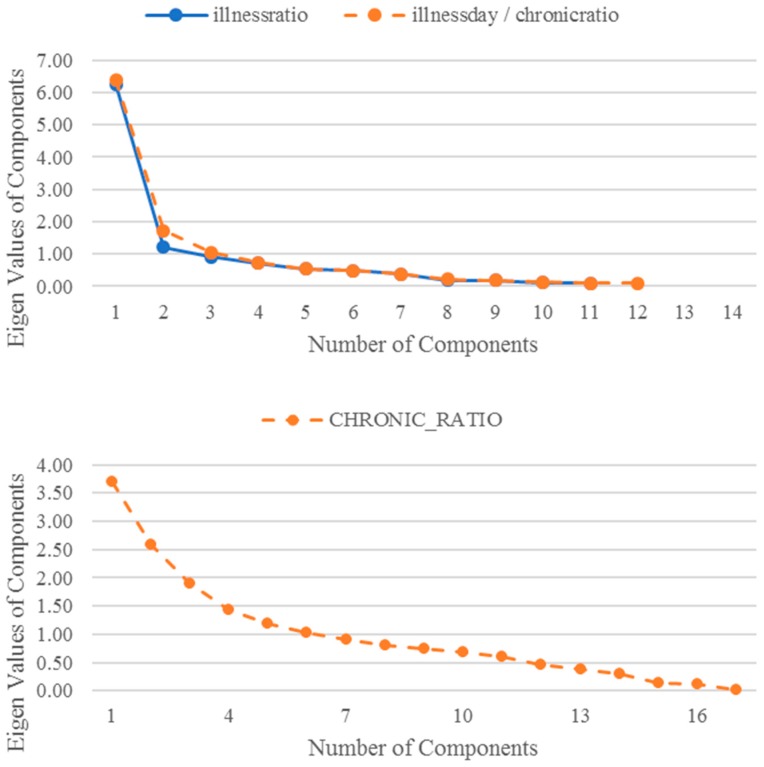
Scree plots.

PCA, as one of the most popular dimensionality reduction techniques, has been widely used to construct socioeconomic status indices [51,52,53,54], because there are many similar variables to collect in surveys, where similar information may be covered by different variables. PCA can eliminate variable duplication, distinguish dimensions of information, and save as much variance as possible while reducing dimensionality [55]. Thus, considering we have many candidate socioeconomic indicator variables with similar but different economic meanings, we use PCA to summarize these variables to several interpretable components. These components, rather than original socioeconomic indicator variables, are used as control variables in our final model specifications. Specifically, we perform the regression-based PCA with maximum variance on the centralized and scaled socioeconomic indicator variables. Components are selected according to their eigen values (greater than or equal to 1). Finally, these components are named according to loading matrices. In Appendix B, Figure A1 reports the scree plots, when Table A3, Note: 1. Loadings are colored by column. Larger correlations have deeper red colors.

Table A4, and Note: 1. loadings are colored by column. Larger correlations have deeper red colors.

Table A5 display the loading matrices with component names.

The final model specifications for different health outcomes are:

$$\begin{array}{ll} illnessratio_{it} & =income_{it}+income2_{it}+edu_{it}+urbanization_{it}+medical\;burden_{it}+geographic\;accessibility_{it}\\ & +\varepsilon_{it} \end{array}$$

$$\begin{array}{ll} illnessday_{it} & =income_{it}+income2_{it}+edu_{it}+urbanization_{it}+medical\;burden_{it}+geographic\;accessibility_{it}\\ & +health\;insurance_{it}+\varepsilon_{it} \end{array}$$

$$\begin{array}{ll} chronicratio_{it} & =income_{it}+income2_{it}+edu_{it}+urbanization_{it}+medical\;burden_{it}+geographic\;accessibility_{it}\\ & +health\;insurance_{it}+\varepsilon_{it} \end{array}$$

$$\begin{array}{ll} CHRONIC\_RATIO_{it} & =AVGINDIINCOME\_EARN_{it}+AVGINDIINCOME\_EARN2_{it}+AVGEDU_{it}+children\;support_{it}\\ & +family\;relations_{it}+consumption_{it}+physical\;burden_{it}+medical\;burden_{it}+drinking_{it}\\ & +unemployment_{it}+\varepsilon_{it} \end{array}$$

## 3. Results

In the regression analysis on both the NHSS and CHARLS datasets, we use feasible generalized least square (FGLS) estimators to avoid possible heteroscedasticity problems. Meanwhile, in addition to individual fixed effect model and individual random effect model, three kinds of regressions are also reported as robustness check: Two-ways fixed effect models are used to exclude the effect of time in short panel datasets; pooling models with FGLS estimator are used to see whether individual effects significantly affect estimation results; and pooling models with OLS estimator are reported as the most conservative estimates. Table 4 displays the main results of the regression analysis of the county-level NHSS dataset, and Table 5 reports the main results of the analysis of the city-level CHARLS dataset. Meanwhile, in Appendix C, we provide complete regression results without effect terms.

Table 4 presents the estimates of the effects of income per capita, squared income per capita and education years on three kinds of morbidities. Every column summarizes the result of a specific model, where the standard errors of coefficients are reported in parentheses. In regressions on the three morbidities, the squared income per capita has significant positive coefficients whose estimates are robust among different models. Education years, however, do not display a statistically significant impact on all the three dependent variables. Meanwhile, the income per capita shows a significant negative effect on the prevalence of chronic diseases, while it does not show such an impact on two-week incidence rate and the number of sick days per thousand people.

The coefficients of squared income per capita indicate non-linear correlations between income per capita and our three kinds of morbidities: two-week prevalence, the number of sick days and chronic disease prevalence. Table A6 in Appendix C displays the variance inflation factor (VIF) whose values are less than 5. The VIF excludes the possibility that the significance of the coefficient of squared income per capita is a fake one raised by the collinearity with the linear term (*income*). When correlations can be profiled with quadratic curves, it means that there are turning-point levels of income per capita: when people’s income is lower than these levels, the three health outcome indicators decline with the growth of income level; however, when people’s income is higher than the turning-point levels, the increase of incidence and prevalence is positively correlated with income per capita. In this context, the linear term of income per capita (*income*) does not solely reflect income’s correlation with morbidity, but its coefficient decides the turning-point level of income per capita together with the coefficient of squared income per capita. However, in our analysis on two-week incidence rate and the number of sick days per thousand people, the specific turning-point level of income per capita cannot be determined, because the coefficients of the linear term of income per capita are not statistically significant but also not robust among different models.

When age and other socioeconomic indicators controlled, the analysis on the CHARLS dataset presents similar estimation results: the coefficient of squared earned income per capita is significantly positive among different models; the average education years has no robust significant impact on the prevalence of chronic diseases. Nevertheless, the coefficient of the linear term of earned income per capita becomes significantly negative in the analysis on the CHARLS dataset. Therefore, we estimate that the turning-point level of earned income per capita locates in the range from 11.1 thousand yuan to 12.1 thousand yuan.

The regression analysis on the NHSS and CHARLS datasets do not fully support the conclusions of descriptive statistics: a non-linear correlation between morbidities and income per capita was discovered; and education, however, was found to have no significant impact on morbidities. The quadratic relationship between income per capita and morbidities is a new answer to the argument whether there is a universal income—morbidity correlation in China. So far, different correlations have been discovered in different countries: negative correlation was found in the U.S. and Europe [9,33,46], while no specific correlation was found in Canada [4]. Meanwhile, a study in Europe [9] pointed out that the negative income—morbidity correlation in Europe is non-linear among different income strata. Thus, what is the case of China? In our analysis, both positive and negative income—morbidity correlations are found in China, where the relationship is also found to be non-linear: morbidity decreases with growing per capita income; however, when income per capita exceeds a specific turning-point level, morbidity begins to increase with continuing income growth. This non-linear relationship in China can be profiled with a quadratic curve. Therefore, this paper suggests distinguishing different income groups when discussing the relationship between income and morbidity in China, e.g., designing gradient contribution policies of the health insurance plans in China. Other than the effect of income per capita on morbidity, the effect of education years in our analysis is consistent with previous research conducted in China [36], i.e., education years have no significant effect on morbidity in different cohorts. However, a negative correlation between education and morbidity is found in the older population in other countries, e.g., the U.S. [33]. This paper does not discuss the reasons for this difference. It should be discussed with causality analysis.

## 4. Conclusions

This study focuses on the relationship between socioeconomic status and the morbidity rate in China. It concerns the cohorts at not only all age stages but also old age stages to add new evidence for the fragmentary relationship between socioeconomic status and morbidity. In our regression analysis on the NHSS and CHARLS datasets, three morbidities are used as dependent variables: two-week incidence rate, the number of sick days per thousand people, and the prevalence of chronic diseases. Meanwhile, we use PCA to convert different socioeconomic indicator variables to several interpretable components as the controlling variables in our model specifications. Then, these specifications are estimated with five models, where our robustness check shows consistent estimates among different models.

The quadratic relationship between income per capita and morbidities were found in both the NHSS and CHARLS data. This relationship is statistically robust on different models and on all the three dependent variables. Such a non-linear correlation means there is an all-age quadratic pattern between the income and morbidities in China. This correlation is similar to the patterns in European countries [9] and England [32]. Meanwhile, our study found no correlation between the education level and the two-week incidence rate in both all-age and old-age cohorts in China. The same conclusion was also found on the number of sick days and chronic disease prevalence in the NHSS data, also the chronic disease prevalence in the CHARLS data. Our conclusions indicate that the relationship between education and morbidity rates in China is consistent to the cases in Canada [4], England [32], and Nordic countries [34]. Such a relationship is different from the cases in the United States [33] and European countries [9,35], where previous research found that education is associated with the onset of health problems.

However, the average education level may visibly affect the correlation between education and morbidity rates. Previous studies suggest that education affects morbidity rates through people’s medical knowledge. However, the average education years is about 7 years in NHSS data and 2 years in CHARLS data, where CHARLS interviews people over 45 years old in China. These respondents were all born no later than 1970 and received less education than the younger generations in China. It suggests that the low education level and generally insufficient medical knowledge of this cohort may be one of the causes of absent association between education level and morbidity in China.

In summary, the negative correlation between socioeconomic status and morbidity might not be proved to be a general pattern, but instead depends on the studied countries. In this paper, the relationship between socioeconomic status and morbidity in China was proved to be not universally negative, which is similar patterns to the cases in some other countries

## Figures and Tables

**Table 1:** Variables from *National Health Services Survey* (NHSS) Data.

	Variables	Codes	Unit	Variable Explanation
Dependent variable	Two-week incidence rate	*illnessratio*	%	Number of injuries per two weeks in every 100 respondents
	Prevalence of chronic diseases	*chronicratio*	%	Number of chronic illness cases in every 100 years of age 15 and over
	Number of sick days per thousand people	*illnessday*	day	Average number of sick days in two weeks per 1000 people
Socioeconomic characteristics	Income per capita	*income*	10 thousand	Average annual income per capita
	Average years of education	*edu*	year	The years required by a respondent to obtain his/her highest degree
Demographic characteristics	Average age in county	*average*	age	The average age of all age group weighted by group size
	Older population proportion	*age65*	%	Proportion of the population over 65 years old
	Male population proportion	*male*	%	Proportion of male population
	Urban flag	*urban*		Urban = 1, rural = 0
Consumption and health service characteristics	Annual consumption	*expend*	yuan	Total annual consumption per capita
	Average of medical treatment costs	*permedicost*	yuan	Average annual medical treatment costs
	Average hospitalization expense	*perhospitalcost*	yuan	Average hospitalization expense of each time
	Health expenditure per capita	*medicost*	yuan	Proportion of family health expenditure to total living expenses
Accessibility and affordability of health services	Accessibility of distance to the nearest hospital	*distance*	%	Proportion of the population whose distance from the nearest hospital to their home is less than 1 km
	Accessibility of time to the nearest hospital	*time10*	%	Proportion of the population whose time cost to the nearest hospital is less than 10 min
	Coverage of social health insurance plans	*insurance*	%	Proportion of the population covered by social health insurance plans
Environment factor	Hygienic toilets shares	*washroom*	%	Proportion of hygienic toilets

**Table 2:** Descriptive statistics.

Variables	Mean	Stdev	Min	Pct 25%	Median	Pct 75%	Max
NHSS (282 observations in 3 years)							
*illnessratio*	16.35	7.53	3.71	11.43	14.70	19.29	53.20
*illnessday*	1328.88	691.82	231.00	854.75	1167.50	1589.25	4128.00
*chronicratio*	14.14	6.38	2.89	9.84	12.97	17.97	33.55
income *	0.00	2.56	−4.98	−1.57	−0.64	1.14	11.07
*income2*	6.54	15.14	0.00	0.61	1.97	5.80	122.54
*edu*	7.39	1.91	1.70	6.15	7.00	8.74	11.65
CHARLS (378 observations in 3 years)							
*CHRONIC_RATIO*	0.75	0.10	0.45	0.68	0.75	0.82	0.98
*AVGINDIINCOME_EARN*	0.44	0.32	0.03	0.21	0.35	0.59	1.81
*AVGINDIINCOME_EARN2*	0.30	0.47	0.00	0.05	0.12	0.35	3.29
*AVGEDU*	1.17	0.16	1.00	1.07	1.12	1.20	1.94

* The original income variable has been regressed with education years. Its statistics in this table are based on the regression residuals.

**Table 3:** Gini coefficients of health outcomes.

**NHSS**				
**Year**	**Counties**	**Illnessratio**	**Illnessday**	**Chronicratio**
1998	94	0.2316	0.2512	0.2790
2003	95	0.1998	0.2395	0.2312
2008	93	0.2514	0.2837	0.2196
**CHARLS**				
**Year**	**Cities**	**CHRONIC_RATIO**		
2011	126	0.0744		
2013	126	0.0640		
2015	126	0.0511		

**Table 4:** Results of the regression analysis on NHSS dataset.

Variable	Individual Fixed Effect (FGLS) ⱡ	Individual Random Effect (FGLS) ⱴ	Two-way Fixed Effect (FGLS) ⱡ	Pooling (FGLS)	Pooling (OLS)
	*illnessratio*				
*income*	−0.3118	0.1051	−0.5214	−0.2278	−0.2278
	(0.3252)	(0.3149)	(0.3371)	(0.3391)	(0.3391)
*income2*	0.2167 ***	0.1758 ***	0.2243 ***	0.1756 ***	0.1756 ***
	(0.0379)	(0.0387)	(0.0378)	(0.0395)	(0.0395)
*edu*	−0.1521	0.6774	−0.4592	0.2953	0.2953
	(0.5851)	(0.5469)	(0.5938)	(0.5557)	(0.5557)
	*illnessday*				
*income*	−13.7166	3.3721	−26.7318	−34.9995	−34.9995
	(28.7490)	(27.7268)	(30.0394)	(30.0065)	(30.0065)
*income2*	18.7594 ***	16.8128 ***	19.0379 ***	17.7110 ***	17.7110 ***
	(3.3440)	(3.3296)	(3.3100)	(3.4914)	(3.4914)
*edu*	−20.0401	62.9851	−37.8590	19.5179	19.5179
	(53.0576)	(48.5439)	(52.7612)	(49.5975)	(49.5975)
	*chronicratio*				
*income*	−0.5160**	−0.4471**	−0.5789**	−0.7147 ***	−0.7147 ***
	(0.2219)	(0.2074)	(0.2308)	(0.2249)	(0.2249)
*income2*	0.0865 ***	0.0789 ***	0.0868 ***	0.0974 ***	0.0974 ***
	(0.0258)	(0.0246)	(0.0254)	(0.0262)	(0.0262)
*edu*	−0.3765	0.0614	−0.4943	−0.1397	−0.1397
	(0.4096)	(0.3752)	(0.4054)	(0.3717)	(0.3717)

Note: 1. Standard errors are reported in parentheses. 2. Models marked with ⱡ use province-level individual fixed effects. Models marked with ⱴ use city-level individual random effects. 3. *** *p* < 0.01, ** *p* < 0.05.

**Table 5:** Results of the regression analysis on *China Health and Retirement Longitudinal Study* (CHARLS) dataset.

Variable	Individual Fixed Effect (FGLS) ⱡ	Individual Random Effect (FGLS) ⱴ	Two-way Fixed Effect (FGLS) ⱡ	Pooling (FGLS)	Pooling (OLS)
	CHRONIC_RATIO				
*AVGINDIINCOME_EARN*	−0.1982 ***	−0.1724 ***	−0.1482 ***	−0.2609 ***	−0.2609 ***
	(0.0452)	(0.0342)	(0.0433)	(0.0493)	(0.0493)
*AVGINDIINCOME_EARN2*	0.0882 ***	0.0792 ***	0.0670 ***	0.1081 ***	0.1081 ***
	(0.0253)	(0.0187)	(0.0239)	(0.0285)	(0.0285)
*AVGEDU*	−0.0768 **	0.0221	−0.0509	0.0338	0.0338
	(0.0362)	(0.0405)	(0.0340)	(0.0379)	(0.0379)

Note: 1. Standard errors are reported in parentheses. 2. Models marked with ⱡ use province-level individual fixed effects. Models marked with ⱴ use city-level individual random effects. 3. *** *p* < 0.01, ** *p* < 0.05.

## Appendix A: Variables and other descriptive statistics

**Table A1:** Variable definitions of CHARLS (complete).

Variables	Codes	Unit	Variable explanation
Prevalence of chronic disease	*CHRONIC_RATIO*	prop	Proportion of the respondents who have at least one of the 13 kinds of chronic diseases
1-year earned incomes per capita	*AVGINDIINCOME_EARN*	10-thousand yuan	Annually incomes from employment per capita
Square of the 1-year earned incomes per capita	*AVGINDIINCOME_EARN2*	10-thousand yuan square	Square of *AVGINDIINCOME_EARN*
Average education years	*AVGEDU*	year	Approximate education years
Average age	AVGAGE	year	Average age
Marriage rate	MARITAL_RATIO	prop	Ratio of married respondents
Marriage years	MARITAL_AVELEN	year	Years of current or the latest marriage
Drinking rate	DRINK1Y_RATIO	prop	Ratio of the respondents who ever drank in the past 1 year
Smoking rate	SMOKENOW_RATIO	prop	Ratio of smoking respondents
Smoking frequency	AVGSMOKENUM	integer	Number of cigarettes smoked per day
1-year inpatient expenditure	AVGHOSP1Y_REALEXP	yuan	Out-of-pocket inpatient expenditure in the past 1 year
1-month outpatient expenditure	AVGOUTP1M_REALEXP	yuan	Out-of-pocket outpatient expenditure in the past 1 month
1-week food consumption	AVGEXP1W_FOOD	yuan	Food consumptions in the past 1 week
Coverage rate of health insurance	INSURANCE_RATIO	prop	Ratio of the respondents who are covered by any kind of health insurance plans
Coverage rate of public health insurance	INSGOV_RATIO	prop	Ratio of the respondents who are covered by government or public health insurance plans
1-year total consumption	AVGEXP1Y_TOTAL	yuan	Total amount of consumptions in the past 1 year
Children support rate	CHILDCARE_RATIO	prop	Ratio of the respondents who receive any kind of supports from children or grandchildren
Children co-residence rate	CHILDCORESD_RATIO	prop	Ratio of the respondents living with children or grandchildren
Children living-nearby rate	CHILDLVNEAR_RATIO	prop	Ratio of the respondents whose children live nearby
Children financial support rate	TRANSCHILD_RATIO	prop	Ratio of the respondents who receive financial support from children
Working rate	WORK_RATIO	prop	Ratio of the respondents who reported they are still working de facto
Agricultural-work rate	JOBSTATUS_AGRI_RATIO	prop	Ratio of the respondents who reported they are doing agricultural jobs
Non-agricultural employment rate	JOBSTATUS_NAGE_RATIO	prop	Ratio of the respondents who reported they are employed by non-agricultural jobs
Non-agricultural self-employment rate	JOBSTATUS_NAGS_RATIO	prop	Ratio of the respondents who reported they are self-employed by non-agricultural jobs
Unemployment rate	JOBSTATUS_UNEM_RATIO	prop	Ratio of the respondents who reported they are unemployed but not retired yet
Never-worked rate	JOBSTATUS_NEWK_RATIO	prop	Ratio of the respondents who reported they never worked before

Note: “prop” indicates proportions, a digit in the range from 0 to 1.

**Table A2:** Inequality indices (complete).

Index	NHSS					CHARLS		
	Year	Counties	Illnessratio	Illnessday	Chronicratio	Year	Cities	CHRONIC_RATIO
Gini	1998	94	0.2316	0.2512	0.2790	2011	126	0.0744
	2003	95	0.1998	0.2395	0.2312	2013	126	0.0640
	2008	93	0.2514	0.2837	0.2196	2015	126	0.0511
Theil-I	1998	94	0.0874	0.1026	0.1215	2011	126	0.0089
	2003	95	0.0635	0.0909	0.0860	2013	126	0.0065
	2008	93	0.1026	0.1287	0.0763	2015	126	0.0041
Theil-II	1998	94	0.0852	0.1003	0.1286	2011	126	0.0091
	2003	95	0.0670	0.0956	0.0932	2013	126	0.0067
	2008	93	0.0994	0.1275	0.0799	2015	126	0.0041
Coef of Variation	1998	94	0.4418	0.4808	0.5048	2011	126	0.1326
	2003	95	0.3603	0.4360	0.4189	2013	126	0.1141
	2008	93	0.4825	0.5375	0.3977	2015	126	0.0901

## Appendix B: PCA Results

**Table A3:** Component loadings (*illnessratio*).

Components	RC2	RC1	RC3
Names	Medical Burden	Urbanization	Geographic Accessibility
urban	0.1699	0.8565	0.2240
expend	0.7731	0.5070	0.1652
medicost	0.8623	0.2998	0.1742
male	−0.3136	−0.6983	0.0610
age65	0.5439	0.6531	−0.0027
average	0.6634	0.5976	0.1000
time10	0.0881	0.1195	0.9264
permedicost	0.6268	0.2827	0.2826
perhospitalcost	0.6812	0.5051	0.2325
insurance	0.7841	0.0106	−0.1891
washroom	0.1589	0.8759	0.1772

Note: 1. Loadings are colored by column. Larger correlations have deeper red colors.

**Table A4:** Component loadings (*illnessday*/*chronicratio*).

Components	RC3	RC1	RC2	RC4
Names	Urbanization	Medical Burden	Geographic Accessibility	Health Insurance
*urban*	0.8140	0.3303	0.2173	−0.0703
*expend*	0.4763	0.6673	0.1357	0.4397
*medicost*	0.2633	0.7471	0.1083	0.4842
*male*	−0.7305	−0.0573	−0.1031	−0.3919
*age65*	0.6501	0.4076	0.0325	0.3698
*average*	0.5901	0.4928	0.1219	0.4591
*distance*	0.2185	0.0655	0.9288	−0.0077
*time10*	0.0766	0.1292	0.9450	0.0397
*permedicost*	0.1898	0.8772	0.0679	0.0250
*perhospitalcost*	0.4583	0.6838	0.1624	0.2964
*insurance*	0.0677	0.2284	−0.0312	0.8704
*washroom*	0.8474	0.2864	0.1460	−0.0374

Note: 1. loadings are colored by column. Larger correlations have deeper red colors.

**Table A5:** Component loadings (*CHRONIC_RATIO*).

Components	RC5	RC1	RC2	RC3	RC4	RC6	RC7
Names	Children Support	Family Relations	Consumption	Physical Burden	Drinking	Medical Burden	Un−Employment
AVGAGE	−0.2146	0.8811	0.0671	0.0786	−0.0063	0.1024	−0.0350
MARITAL_RATIO	−0.1523	−0.4380	−0.3284	0.1957	0.3946	0.1868	0.2155
MARITAL_AVELEN	−0.1560	0.8706	−0.0073	−0.1443	0.0626	0.0763	0.1515
DRINK1Y_RATIO	−0.2836	−0.0658	0.0067	0.0926	0.7237	−0.0122	−0.1155
AVGHOSP1Y_REALEXP	0.1042	0.2534	0.3165	0.1663	0.1429	0.5617	−0.0350
AVGOUTP1M_REALEXP	−0.1089	−0.0278	0.1028	−0.0954	−0.0570	0.7873	0.1441
AVGEXP1W_FOOD	0.0023	0.0675	0.9361	−0.0117	0.0353	0.1028	0.0147
AVGEXP1Y_TOTAL	0.0320	0.0371	0.9127	0.1526	0.0583	0.1972	−0.0049
CHILDCARE_RATIO	0.9551	−0.1080	−0.0514	0.0988	−0.0629	0.0202	−0.0128
CHILDCORESD_RATIO	0.7243	−0.2237	0.2799	−0.1026	−0.2160	−0.1179	0.0216
CHILDLVNEAR_RATIO	0.9489	−0.1397	−0.0571	0.0848	−0.0784	0.0073	−0.0054
TRANSCHILD_RATIO	−0.2022	0.6391	0.0789	−0.2419	0.2643	0.0540	0.3319
WORK_RATIO	0.0219	−0.1650	−0.1095	−0.5946	0.3037	−0.4071	0.3917
JOBSTATUS_NAGE_RATIO	0.1384	−0.1395	0.0788	0.7640	0.1820	0.0212	−0.1084
JOBSTATUS_NAGS_RATIO	−0.0906	−0.2229	0.0255	0.6041	−0.0895	−0.3563	0.4260
JOBSTATUS_UNEM_RATIO	−0.0371	−0.2112	0.0018	0.0694	−0.0347	−0.1339	−0.7833
JOBSTATUS_NEWK_RATIO	−0.0098	−0.3213	−0.1421	0.0629	−0.6985	0.0012	−0.1844

Note: 1. Loadings are colored by column. Larger correlations have deeper red colors.

## Appendix C: Other Results

**Table A6:** VIF.

NHSS						CHARLS	
Illnessratio		Illnessday		Chronicratio		CHRONIC_RATIO	
*income*	4.857	*income*	4.875	*income*	4.875	*AVGINDIINCOME_EARN*	15.597
*income2*	2.298	*income2*	2.304	*income2*	2.304	*AVGINDIINCOME_EARN2*	11.190
*edu*	7.238	*edu*	7.388	*edu*	7.388	*AVGEDU*	2.210
RC1	3.934	RC1	4.309	RC1	4.309	RC1	1.107
RC2	4.764	RC2	1.881	RC2	1.881	RC2	1.053
RC3	1.992	RC3	3.564	RC3	3.564	RC3	2.815
		RC4	2.091	RC4	2.091	RC4	1.049
						RC5	1.022
						RC6	1.354
						RC7	1.281

Note: 1. Rotated principle components (RCn) of different specifications have the same variables names but distinct definitions, loadings and scores. 2. Blank lines are spared to keep the table equally high among columns.

**Table A7:** Complete regression results (NHSS).

Variable	Individual Fixed Effect (FGLS) ⱡ	Individual Random Effect (FGLS) ⱴ	Two-way Fixed Effect (FGLS) ⱡ	Pooling (FGLS)	Pooling (OLS)
***illnessratio***					
(Intercept)	21.8469 ***	10.0209 **	24.5788 ***	13.0202 ***	13.0202 ***
	(4.4718)	(3.9618)	(4.6590)	(4.0368)	(4.0368)
*income*	−0.3118	0.1051	−0.5214	−0.2278	−0.2278
	(0.3252)	(0.3149)	(0.3371)	(0.3391)	(0.3391)
*income2*	0.2167 ***	0.1758 ***	0.2243 ***	0.1756 ***	0.1756 ***
	(0.0379)	(0.0387)	(0.0378)	(0.0395)	(0.0395)
*edu*	−0.1521	0.6774	−0.4592	0.2953	0.2953
	(0.5851)	(0.5469)	(0.5938)	(0.5557)	(0.5557)
RC2 (medical burden)	1.9514 **	0.6978	2.3577 **	1.7011 **	1.7011 **
	(0.8429)	(0.8103)	(1.0439)	(0.8601)	(0.8601)
RC1 (urbanization)	0.9746	0.0438	1.4062	0.5049	0.5049
	(0.8793)	(0.7792)	(0.8967)	(0.7816)	(0.7816)
RC3 (geographic accessibility)	0.0508	−0.4741	0.4391	−0.1480	−0.1480
	(0.5704)	(0.5538)	(0.5890)	(0.5562)	(0.5562)
***illnessday***					
(Intercept)	1631.8347 ***	744.3945 **	1706.3932 ***	1068.9368 ***	1068.9368 ***
	(401.3076)	(351.3749)	(415.9300)	(360.3742)	(360.3742)
*income*	−13.7166	3.3721	−26.7318	−34.9995	−34.9995
	(28.749)	(27.7268)	(30.0394)	(30.0065)	(30.0065)
*income2*	18.7594 ***	16.8128 ***	19.0379 ***	17.7110 ***	17.7110 ***
	(3.3440)	(3.3296)	(3.3100)	(3.4914)	(3.4914)
*edu*	−20.0401	62.9851	−37.8590	19.5179	19.5179
	(53.0576)	(48.5439)	(52.7612)	(49.5975)	(49.5975)
RC3 (urbanization)	174.3866 **	32.8512	183.2006**	84.5007	84.5007
	(76.2881)	(65.5659)	(75.7176)	(65.7198)	(65.7198)
RC1 (health burden)	54.1368	−59.3752	155.9446 **	55.7788	55.7788
	(69.9565)	(68.4793)	(76.2863)	(72.2603)	(72.2603)
RC2 (geographic accessibility)	34.8607	20.7914	41.7407	38.9836	38.9836
	(52.4659)	(48.0439)	(51.8061)	(47.7470)	(47.7470)
RC4 (health insurance)	171.5954 ***	135.6597 ***	247.2420 ***	200.8171 ***	200.8171 ***
	(49.6029)	(46.1464)	(69.9900)	(50.3339)	(50.3339)
***chronicratio***					
(Intercept)	16.9854 ***	13.0889 ***	16.9771 ***	14.5350 ***	14.5350 ***
	(3.0980)	(2.7301)	(3.1958)	(2.7004)	(2.7004)
*income*	−0.5160 **	−0.4471**	−0.5789 **	−0.7147 ***	−0.7147 ***
	(0.2219)	(0.2074)	(0.2308)	(0.2249)	(0.2249)
*income2*	0.0865 ***	0.0789 ***	0.0868 ***	0.0974 ***	0.0974 ***
	(0.0258)	(0.0246)	(0.0254)	(0.0262)	(0.0262)
*edu*	−0.3765	0.0614	−0.4943	−0.1397	−0.1397
	(0.4096)	(0.3752)	(0.4054)	(0.3717)	(0.3717)
RC3 (urbanization)	4.0814 ***	3.1392 ***	4.1033 ***	3.3727 ***	3.3727 ***
	(0.5889)	(0.5082)	(0.5818)	(0.4925)	(0.4925)
RC1 (health burden)	2.0875 ***	1.6458 ***	2.9994 ***	2.2899 ***	2.2899 ***
	(0.5400)	(0.5160)	(0.5862)	(0.5415)	(0.5415)
RC2 (geographic accessibility)	0.5926	0.5248	0.6255	0.5997 *	0.5997 *
	(0.4050)	(0.3805)	(0.3981)	(0.3578)	(0.3578)
RC4 (health insurance)	2.5506 ***	2.4239 ***	3.3971 ***	2.6387 ***	2.6387 ***
	(0.3829)	(0.3520)	(0.5378)	(0.3772)	(0.3772)

Note: 1. Standard errors are reported in parentheses. 2. Models marked with ⱡ use province-level individual fixed effects. Models marked with ⱴ use city-level individual random effects. 3. *** *p* < 0.01, ** *p* < 0.05, * *p* < 0.1.

**Table A8:** Complete regression results (CHARLS).

Variable	Individual Fixed Effect (FGLS) ⱡ	Individual Random Effect (FGLS) ⱴ	Two-way Fixed Effect (FGLS) ⱡ	Pooling (FGLS)	Pooling (OLS)
***CHRONIC_RATIO***					
(Intercept)	0.9415 ***	0.7798 ***	0.9772 ***	0.7906 ***	0.7906 ***
	(0.0549)	(0.0480)	(0.0515)	(0.0460)	(0.0460)
*AVGINDIINCOME_EARN*	−0.1982 ***	−0.1724 ***	−0.1482 ***	−0.2609 ***	−0.2609 ***
	(0.0452)	(0.0342)	(0.0433)	(0.0493)	(0.0493)
*AVGINDIINCOME_EARN2*	0.0882 ***	0.0792 ***	0.0670 ***	0.1081 ***	0.1081 ***
	(0.0253)	(0.0187)	(0.0239)	(0.0285)	(0.0285)
*AVGEDU*	−0.0768 **	0.0221	−0.0509	0.0338	0.0338
	(0.0362)	(0.0405)	(0.0340)	(0.0379)	(0.0379)
RC5 (children support)	−0.0137 ***	−0.0203 ***	−0.0059	−0.0217 ***	−0.0217 ***
	(0.0039)	(0.0033)	(0.0039)	(0.0041)	(0.0041)
RC1 (family relations)	0.0409 ***	0.0462 ***	0.0090	0.0333 ***	0.0333 ***
	(0.0037)	(0.0032)	(0.0060)	(0.0042)	(0.0042)
RC2 (consumption)	0.0236 ***	0.0163 ***	0.0119 ***	0.0295 ***	0.0295 ***
	(0.0037)	(0.0030)	(0.0039)	(0.0041)	(0.0041)
RC3 (physical burden)	0.0150 **	0.0026	0.0070	0.0074	0.0074
	(0.0065)	(0.0060)	(0.0062)	(0.0068)	(0.0068)
RC4 (drinking)	0.0192 ***	0.0109 ***	0.0107 **	0.0122 ***	0.0122 ***
	(0.0043)	(0.0039)	(0.0042)	(0.0041)	(0.0041)
RC6 (medical burden)	0.0244 ***	0.0148 ***	0.0158 ***	0.0241 ***	0.0241 ***
	(0.0042)	(0.0031)	(0.0041)	(0.0047)	(0.0047)
RC7 (unemployment)	0.0013	0.0043	−0.0051	0.0023	0.0023
	(0.0041)	(0.0031)	(0.0042)	(0.0046)	(0.0046)

Note: 1. Standard errors are reported in parentheses. 2. Models marked with ⱡ use province-level individual fixed effects. Models marked with ⱴ use city−level individual random effects. 3. *** *p* < 0.01, ** *p* < 0.05.

**Table A9:** Normality tests on regression residuals.

	**NHSS**		
	***Illnessratio***	***Illnessday***	***Chronicratio***
Shapiro-Wilk	0.000	0.003	0.091
Lilliefor	0.007	0.002	0.321
Pearson Chi-square	0.321	0.001	0.371
Anderson-Darling	0.001	0.001	0.256
Cramer-von Mises	0.002	0.002	0.295
	**CHARLS**		
	***CHRONIC_RATIO***		
Shapiro-Wilk	0.151		
Lilliefor	0.401		
Pearson Chi-square	0.569		
Anderson-Darling	0.134		
Cramer-von Mises	0.239		

Note: 1. residuals are calculated based on the fixed individual effect models; 2. H0: if *p* > α, the sample is from a normal distribution.
